# Effects of a medical second opinion programme on patients’ decision for or against knee arthroplasty and their satisfaction with the programme

**DOI:** 10.1186/s12891-021-04465-5

**Published:** 2021-06-28

**Authors:** Martin Weigl, Jens Pietzner, Rebecca Kisch, Alexander Paulus, Volkmar Jansson, Eva Grill

**Affiliations:** 1 Department of Orthopaedics, Physical Medicine and Rehabilitation, University Hospital, LMU Munich, Marchioninistr. 15, D-81377 Munich, Germany; 2 Kliniken an der Paar, Aichach und Friedberg, Germany; 3 Institute for Medical Information Processing, Biometrics and Epidemiology, Ludwig-Maximilians Universität München, Munich, Germany; 4 German Center for Vertigo and Balance Disorders, Ludwig-Maximilians Universität München, Munich, Germany

**Keywords:** Knee osteoarthritis, Second opinion, Knee arthroplasty, Decision making, Guidelines

## Abstract

**Background:**

German social legislation gives patients the right to obtain a second opinion before elective surgery and defines quality criteria for reimbursement by statutory health insurances. However, the effects of second opinions before elective surgery are largely unknown. The aim of this study was to evaluate the effects of a second opinion programme in patients recommended for knee arthroplasty.

**Methods:**

The largest statutory health insurance funds in Bavaria offered patients who had been recommended to have knee arthroplasty the opportunity to partake in a second opinion programme which consisted of an in person presentation to an experienced knee surgeon. In this cohort study, consecutive patients from this second opinion programme who signed informed consent were included from 07/10/2016 to 14/02/2020. Data were collected before and after the second opinion visit.

**Results:**

A total of 141 (66%) of 215 patients who presented for a second opinion participated in the evaluation study. The second opinion physician recommended knee arthroplasty to 40% of the patients, later knee arthroplasty if the conditions worsened to 40%, and no knee arthroplasty to 20%. After receiving the second opinion 28 of 56 (41%) undecided patients preferred knee arthroplasty, 14 no knee arthroplasty, 14 remained undecided. Four of 46 patients with a preference for “arthroplasty” changed their decision to “no arthroplasty”, five of 35 patients from “no arthroplasty” to “arthroplasty”. The patients were more confident in their decision according to the decision confidence scale (before: 5.4 ± 3.0; after: 7.8 ± 2.5; *p* < 0.001). They rated their satisfaction with the second opinion programme with a mean grade of 1.35 (± 0.60) (best:1; worst:6). Logistic regression analyses showed that the recommendation of the second opinion physician for joint arthroplasty was associated with the guideline criteria radiological severity of osteoarthritis (*p* = 0.001) and knee-joint-specific quality of life (*p* = 0.041).

**Conclusion:**

The second opinion of an experienced knee surgeon frequently deviates from the initial recommendation for knee arthroplasty. The association of guideline criteria to the second recommendation suggests a high quality of the second opinion. From the patient perspective, the second opinion reduces uncertainties in their treatment decision.

## Background

Knee arthroplasty (KA) is a very common procedure in orthopaedic surgery. It is performed particularly frequently in Switzerland, the United States, Austria and Germany, with more than 200 surgical operations per year per 100,000 inhabitants, while the average in 33 Organisation for Economic Co-operation and Development (OECD) countries is 126 [1] per year per 100,00 inhabitants. Population ageing and increasing levels of obesity are expected to more than double the incidence of KA in many countries by 2050 or even earlier [2–4]. The popularity of KA can be explained by the prospect of large improvements in pain and physical function [5]. However, approximately 20% of patients still complain of pain one year after surgery [6, 7] and are not satisfied with the results. New surgical techniques do not seem to reduce the proportion of unsatisfied patients [8].

One possible reason for unsatisfactory outcomes are weaknesses in the decision-making process before KA. This is supported by large age-adjusted variations in frequencies of KA in Germany at the state and district levels [9]. In Bavaria, for example, the probability of receiving KA is 70% higher than in Berlin. Additionally, a study in the United States found substantial regional variations that could not be sufficiently explained by differences in morbidity [10]. An inadequate decision-making process may be one of the causes of these regional differences.

The German S2k guideline indications for KA (2 k = structured consensus) of the Association of the Scientific Medical Societies in Germany (AWMF) recommends that the decision for KA should be based on both medical criteria and patient preferences [11]. It defines five main criteria: knee pain for at least 3–6 months; evidence of structural damage; failure of conservative treatment for at least 3–6 months; limitation of quality of life related to knee joint disease; and subjective substantial suffering. After a physician has determined the indication for KA, the guideline additionally recommends a shared decision-making process. The German AWMF osteoarthritis S2k guideline additionally recommends that if he patient has any doubt, a second medical opinion (SO) from an experienced knee surgeon from a KA centre should be obtained [12]. However, the regional differences suggest that decisions for KA sometimes do not correspond to the recommendations of the guidelines.

Since 2015 patients in the German health care system have the legal right to obtain an independent SO for certain elective surgeries for which, “particularly in view of the numerical development of its implementation, the risk of an expansion of indications cannot be ruled out “(§ 27b, Code of Social Law V) [13]. Social law defines two main quality criteria for the second opinion physician (SP): first, they should have many years of experience as a specialist in a field relevant to the indication for surgery and secondly, they should have knowledge of the current state of scientific research on the relevant diagnostics and therapy, including knowledge of alternative treatments to the recommended intervention. The Federal Joint Committee (G-BA) defines further details in a directive [14]. Statutory health insurance reimburses SO. In October 2020, the G-BA has decided adding KA to the previously existing list of interventions (tonsillectomy, hysterectomy and shoulder arthroscopy) qualifying for a SO [14, 15]. There is, however, still a lack of published evidence confirming the effects of a SO with personal presentation in patients with KA.

In a representative survey in Germany, 56% of the respondents considered it important to have the opportunity to obtain an SO before orthopaedic surgery [16]. However, the literature on the effects of an SO is limited [17, 18]. It therefore remains unclear whether an SO actually improves adherence to guidelines and offers more certainty to patients. In contrast, an SO may increase uncertainty if the recommendations of the initial physician (IP) and the SP are divergent. From the economic perspective, one recent study suggests that an SO has the potential to reduce costs. The study, however, does not enable firm conclusions concerning the cost effectiveness of an SO in patients before KA due to its explorative study design and heterogenic patient population. The study included patients with various diseases of the spine, shoulder, hip, knee, foot and hand [19]. Overall, more evidence concerning the various effects of an SO before KA is required.

In a pilot project, the German statutory health insurance AOK Bayern in cooperation with the Department for Orthopaedics, Physical Medicine and Rehabilitation (OPMR) at the University Hospital Munich provides a patient-initiated SO programme for patients recommended for KA by their IP. The overall objectives of this study were to evaluate this SO programme with in person presentation to an experienced knee surgeon with regard to agreement of recommendations, effects on the patients’ decision for or against KA, and patient satisfaction.

## Methods

### Aims

The primary specific aims were a) to evaluate the agreement between the recommendations of the IP and the SP and b) to examine the effects of the SO on the patients’ decision and the certainty of the decision.

Furthermore, the study aimed to assess the quality of the SO programme. As first criterion for good quality, we defined that the recommendation of the SP should be associated with the main criteria for KA in the German AWMF guideline indications for KA [11]. The second quality criterion was the patients’ satisfaction with the SO concept.

### Study design

This prospective cohort study evaluated an SO pilot project in patients with recommendations for KA by their IP. It was approved by the institutional review board at the medical faculty of the Ludwig Maximilian University Munich (project number 17–098). Written informed consent was obtained from all patients prior to enrolment. The study was conducted in accordance with the Declaration of Helsinki. The analysis plan was registered in the open science framework before the analyses were performed (https://osf.io/ng4fb/).

### Setting

The study was conducted at OPMR, University Hospital, LMU, Munich. OPMR is certified by Endocert as an endoprosthesis centre of maximum care. Endocert is the world’s first joint arthroplasty-specific quality assurance system for certifying the quality of knee and hip arthroplasty. It had certified 543 in German facilities by the end of 2018 [20].

OPMR had a contract with the statutory health insurance fund “AOK Bayern”. AOK Bayern reimbursed OPMR for the costs of the SO visit. There were no incentives from the AOK Bayern for disagreeing with the initial recommendation for KA.

### Patient recruitment and inclusion criteria

Patients were informed about the SO project via the AOK Bayern webpage, the AOK Bayern Facebook page, articles in the AOK members’ magazine and by AOK Bayern branches. In addition, the AOK informed patients who contacted an AOK branch by phone. With over 3.5 million members, AOK Bayern has a market share of more than 40% among the statutory health insurance funds in Bavaria [21].

Interested patients called the OPMR. A trained doctoral student or study nurse reviewed the inclusion criteria. These were: medical insurance with the AOK Bayern, previous recommendation for primary KA (unikondylar or bikondylar), no previous recommendation for cartilage transplantations, no previous recommendation for arthroscopic meniscus surgery and no “knee pain of unknown origin”. Patients fulfilling these criteria were given a SO appointment.

**Fig. 1:**
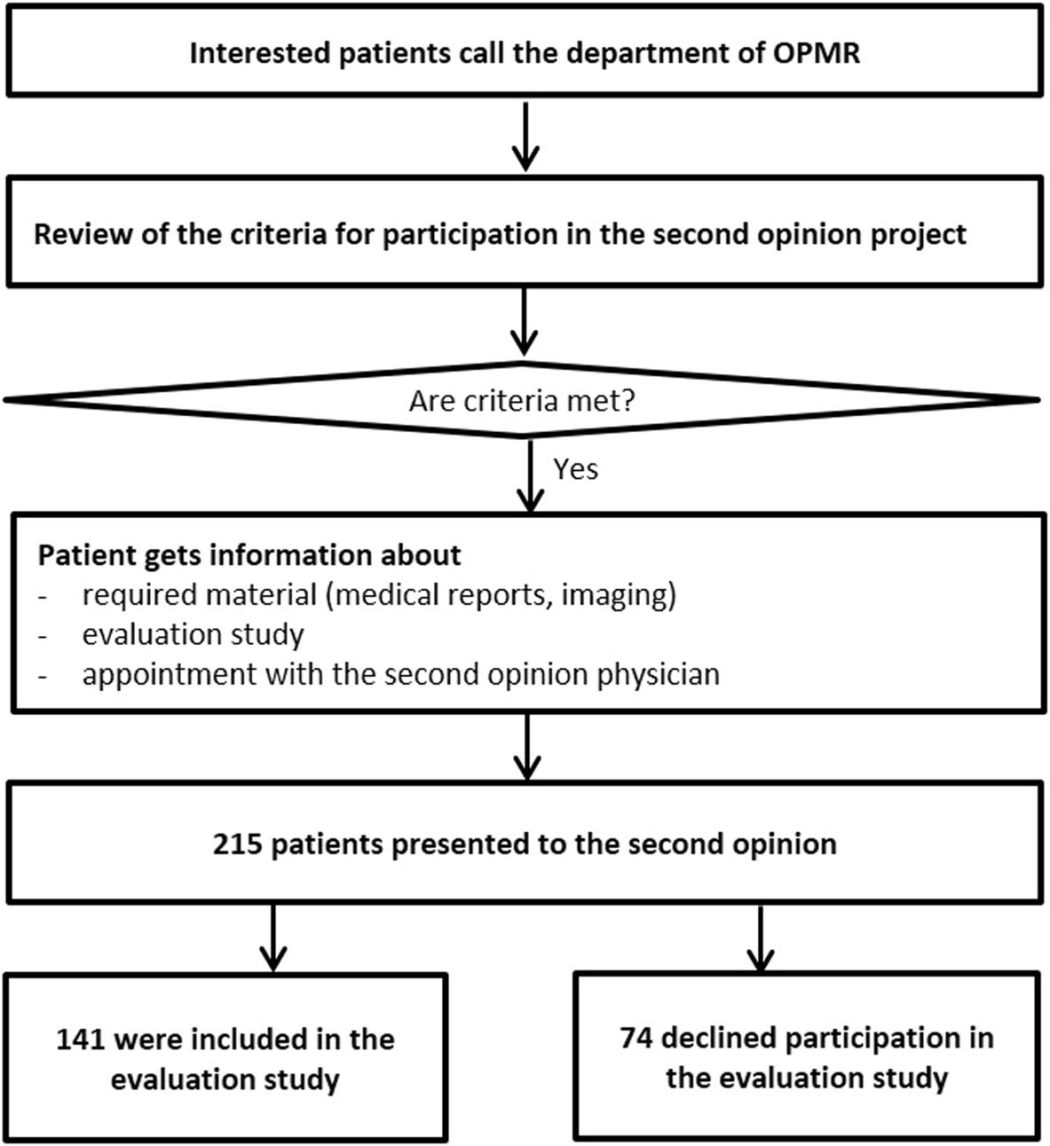
Patient flow

Further inclusion criteria for participation in the evaluation study were a previous x-ray image of the knee in two planes in the last 6 months, sufficient German language skills to fill in questionnaires and a signed informed consent. Figure 1 present the patient flow.

### Intervention

The patients presented in person to the SP. The SP was a specialist in orthopaedics with at least 5 years of experience in KA. The SP evaluated the indication for KA by taking a medical history, performing a clinical examination, evaluating X-ray images and, if available, evaluating other medical reports.

In the medical history, the SP asked particularly about pain during physical activity, at rest and at night, restrictions in daily life, quality of life, pharmacological and non-pharmacological treatments, previous surgery, subjective suffering, previous illnesses, psycho-social stress situations, contraindications for KA and risk factors for surgery. The SP examined the mobility and stability of the knee joint, crepitus, leg axis, clinical signs of inflammation and pain on palpation of the knee joint structures. Depending on the medical history and symptoms, additional examinations were added. The SP assessed the radiological severity of osteoarthritis according to the Kellgren and Lawrence (K-L) scale (see section measures) [22, 23].

At the end of the appointment, the SP discussed the recommendation with the patient, taking into account the strength of the recommendation, the benefits and risks of KA and other treatment options. In recommending KA, the SP aimed to follow the criteria of the German guideline indication for KA [11]. The SP provided the patients with a report that included information on the results of the clinical examination, the evaluation of imaging, and the treatment recommendation.

### Data collection

T0: Participants received a set of questionnaires on the day of the appointment and completed the questionnaires before visiting the doctor. During the visit, height and weight were measured, and X-ray images were evaluated.

T1: Immediately after the visit to the doctor, patients completed a second questionnaire and submitted it on the same day as the examination.

### Measures

The patient decision at T0 and T1 was evaluated by the question “Do you want surgical treatment for your knee?” Patients answered on a 5-point Likert scale: “Yes, definitely”, “rather yes”, “undecided”, “rather no” and “No, definitely not”. Decision confidence was assessed by the question “On a scale from 0 (not at all) to 10 (extremely), how confident are you about your decision for surgery?” These two questions were adapted from a longer decision quality instrument and translated into German. The answer options of the first questions were expanded from a 3-point to a 5-point Likert scale in comparison to the original publication [24].

Pharmacological and non-pharmacological conservative treatment, previous surgeries, comorbidity and sociodemographic data were collected by using closed questions.

The Knee injury and Osteoarthritis Outcome Score (KOOS) was used to measure knee pain (9 items), other knee symptoms (7 items), restrictions in activities of daily living (17 items), restrictions in sport and recreation function (5 items) and knee-related quality of life (4 items) [25–27]. Each item was scored on a 4-point Likert scale. The scale ranged from 0 (worst) to 100 (best). For ease of interpretation of the regression model, the original scale was reversed prior to analysis (0 = best; 100 = worst). The KOOS has been validated in many languages, including German, and has demonstrated good reliability and responsiveness in patients with knee osteoarthritis and KA. Compared to the older, frequently used Western and Ontario Osteoarthritis Index (WOMAC), the KOOS has the advantage of fewer ceiling effects [27].

Generic health status was assessed by the 5-level version of the EuroQOL Group 5-Dimension Self-Report Questionnaire (EQ-5D-5L) [28, 29]. It comprises five items that evaluate five dimensions: mobility, self-care, usual activities, pain/discomfort, and anxiety/depression [23, 24]. An algorithm was used to calculate the EQ-5D-5L index. The range was from − 0.661 (worst health) to 1 (best health). We also applied the EQ-5D-visual analogue scale (EQ-5D-VAS) (0 = worst; 100 = best). The EQ-5D-5L has been validated in patients with knee osteoarthritis and KA [29].

Anxiety and depression were assessed by the Patient Health Questionnaire 4 (PHQ-4) [30]. The PHQ-4 is an ultra-brief, reliable and valid instrument with two items regarding anxiety and depression. Each item has four answer options (score 0–3). The scales range from 0 to 6. Scores ≥3 are considered probable cases of anxiety or depression.

In the course of the study, we added a question on the urgency of the recommendation of the IP because patients provided very different information about the period in which the IP recommended knee surgery to them. Accordingly, a more detailed description of the recommendation was considered important for a better understanding of differences between the IP and the SP. For the categorization of the urgency, we adapted the phrases that were often used by the patients in the first phase of the study: “fixed date of surgery”, “as soon as possible”, “can wait a few more months”, “if condition is worsening” and “at some point of time”.

The radiological severity of osteoarthritis was assessed by the K-L scale [22]. The K-L scale is a commonly used system that classifies the radiological severity of osteoarthritis from 0 to 4 depending on joint space narrowing, osteophytes, sclerosis, and joint deformity of bone ends. Patients with grade 3 or 4 osteoarthritis show larger effects after KA than patients with lower grades [23].

At T1, patients were asked about the recommendation of the SP: “Did your second opinion doctor recommend KA?” The answer options were yes (surgery recommended immediately or within less than 3 months), no or “later surgery recommended depending on the course of the disease”.

To assess the influence of the SO on the patient’s decision, we asked the following question: “How strongly does the second opinion influence your decision for or against a knee prosthesis?” The patients responded on a 5-level Likert scale (min = 1, max = 5): very strongly/strongly/somewhat/little/very little.

Satisfaction with the SO project was measured by the question: “What school grade do you give the AOK-LMU second opinion project?” The response options were adapted from the grading system of German schools: 1 = very good; 2 = good; 3 = satisfying; 4 = sufficient; 5 = deficient; 6 = insufficient.

### Statistical analyses

Details of the statistical analysis plan were made publicly available before analyses were performed (https://osf.io/ng4fb/). The person who conducted the analysis (second author) was not involved in the data collection and was not employed at the SO department. Means and standard deviations were calculated for metric variables. Frequencies are expressed as percentages.

Changes in the frequencies of decision preferences to T0 and T1 were tested for significance using the Chi-square test, and changes in decision confidence were tested for significance using the t-test for paired samples.

The association between the recommendation of the SP and the indication criteria of the German S2k guideline indications for KA was first analysed by descriptively comparing these criteria between patients with and without a current recommendation for KA. The influence of these criteria on the recommendation was then analysed in uni-variable and multivariable logistic regression models. The independent variables were the K-L scale after transformation into a binary scale (1–2 versus 3–4), the KOOS scales for knee pain and knee-joint-related quality of life and previous treatments with exercise therapy (yes/no) or pain medication (no/on demand/always). The cut-off value for the K-L scale was set between 2 and 3 because patients with grade > 2 osteoarthritis show larger treatment effects [23]. The model was further adjusted for age and gender. The dependent variable was the current recommendation for KA (yes/no). We did not use statistical selection of criteria because the model was designed to compare all variables that represent main criteria from the German S2k guideline. The Hosmer-Lemeshow test was used as the goodness-of-fit test.

Statistical analyses were performed with the software package IBM SPSS Statistics for Windows, Version 25.0. Armonk, NY: IBM Corp.

## Results

### Study population

A total of 215 patients met the inclusion criteria for the study between 07/10/2016 and 14/02/2020. Of these, 141 (66%) declared their consent to participate in the study. The sociodemographic data and comorbidities are presented in Table 1. The mean age was 64.5 ± 9.9 years, 70 patients (50%) were female, and 48 patients (36%) were obese with a BMI > 30. Non-responders did not differ from participants (mean age 63.9 ± 11.7; 51% were female).

**Table 1:** Baseline characteristics of the patients

Characteristic	All patients (*n* = 141)	Second opinion: Knee arthroplasty recommended (*n* = 57)	Second opinion: Knee arthroplasty currently not recommended (*n* = 84)
	n (%) or mean ± SD	n (%) or mean ± SD	n (%) or mean ± SD
Female sex (*n* = 141)	70 (50%)	29 (51%)	41 (49%)
Age - yr (*n* = 141)	64.5 ± 9.9	65.8 ± 8.8	63.6 ± 10.5
Living alone (*n* = 141)	31 (22%)	13 (23%)	18 (21%)
Education, highest degree (*n* = 137)			
No degree	2 (2%)	0 (0%)	2 (3%)
Basic school (8–9 years of education)	80 (58%)	35 (63%)	45 (56%)
Middle school (10 years of education)	38 (28%)	15 (27%)	23 (28%)
High school (12–13 years of education)	7 (5%)	3 (5%)	4 (5%)
University	10 (7%)	3 (5%)	7 (9%)
Number of comorbidities (*n* = 141)			
0	20 (14%)	10 (18%)	10 (12%)
1	38 (27%)	13 (23%)	25 (30%)
2	34 (24%)	17 (30%)	17 (20%)
3	23 (16%)	10 (18%)	13 (16%)
≥ 3	26 (18%)	7 (12%)	19 (21%)
Body weight (*n* = 134)			
Body mass index^a^ (BMI)	28.8 ± 5.7	29.4 ± 6.4	28.4 ± 5.0
Normal weight (BMI < 27)	50 (37%)	20 (38%)	30 (37%)
Overweight (BMI 27–30)	36 (27%)	13 (25%)	23 (28%)
Obesity (BMI > 30)	48 (36%)	19 (37%)	29 (35%)
Mental health (PHQ-4)			
Depression score (*n* = 134)	0.91 ± 0.80	0.90 ± 0.78	0.91 ± 0.81
Probable cases of depression	4 (3%)	1 (2%)	3 (4%)
Anxiety score (*n* = 133)	0.65 ± 0.72	0.61 ± 0.75	0.69 ± 0.70
Probable cases of anxiety	4 (3%)	2 (4%)	2 (3%)

^a^The body mass index is the weight in kilograms divided by the square of the height in metres. Patient health questionnaire 4 (PHQ-4): Scores range from 0 to 6. Scores above 3 are considered probable cases of anxiety or depression

Table 2 shows the results of the radiological and patient-relevant outcome measures and the previous treatments. The radiological K-L score was 3 or 4 in 108 patients (77%). Previous exercise therapy was reported by 96 patients (76%), 9 patients (7%) had tried to lose weight, and 39 patients (28%) took pain medication regularly.

**Table 2:** Health status and previous treatments

Characteristic	All patients (n = 141)	Second opinion: Knee arthroplasty recommended (*n* = 57)	Second opinion: Knee arthroplasty currently not recommended (*n* = 84)
	n (%) or mean ± SD	n (%) or mean ± SD	n (%) or mean ± SD
Health Status			
Kellgren-Lawrence score^a^ (*n* = 141)			
1	7 (5%)	0 (0%)	7 (8%)
2	26 (18%)	2 (4%)	24 (29%)
3	69 (49%)	30 (53%)	39 (46%)
4	39 (28%)	25 (44%)	14 (17%)
KOOS^b^ scores			
Symptoms (*n* = 138)	46.5 ± 19.6	53.8 ± 17.2	49.6 ± 17.6
Pain (*n* = 136)	51.3 ± 17.5	49.3 ± 20.5	44.8 ± 19.0
Activities of daily living (*n* = 141)	44.6 ± 19.1	48.7 ± 19.2	41.8 ± 18.5
Sport and recreation (*n* = 127)	72.8 ± 21.3	78.5 ± 18.4	68.9 ± 22.3
Quality of life (*n* = 140)	71.4 ± 15.9	75.2 ± 14.0	68.8 ± 16.7
EQ-5D scores^c^			
Visual analogue scale (*n* = 136)	60.1 ± 18.8	57.4 ± 18.4	61.9 ± 19.1
Index (*n* = 136)	0.64 ± 0.27	0.59 ± 0.29	0.68 ± 0.26
Previous treatment			
Exercise therapy^d^	96 (76%)	43 (83%)	53 (72%)
Attempt to lose weight	9 (7%)	4 (8%)	5 (7%)
Pain medication			
None	34 (24%)	14 (25%)	20 (24%)
On demand	68 (48%)	23 (40%)	45 (54%)
Regularly	39 (28%)	20 (35%)	19 (23%)
Knee surgery	83 (59%)	33 (58%)	50 (60%)
Knee injection	51 (36%)	21 (37%)	30 (36%)

^**a**^Kellgren-Lawrence score: range from 0 to 4, with a score of 2, 3, or 4 indicating definite osteoarthritis and higher scores indicating more severe disease. ^**b**^KOOS: Knee Injury and Osteoarthritis Outcome Score, scores range from 0 (best) to 100 (worst); ^**c**^EQ-5D scores: the visual analogue scale ranges from 0 (worst) to 100 (best); the index score ranges from −0.661 (worst) to 1 (best). ^**d**^Exercise therapy: supervised strength or endurance training (individually or in group) or home-based strength or endurance training or comprehensive rehabilitation intervention

### Agreement of the recommendations between the IP and SP

The SP recommended KA for 57 patients (40%) immediately, later KA if the condition worsened for 56 patients (40%), and no KA for 28 patients (20%).

Table 3 presents the agreement between the urgency of the recommendation of the IP and the SP that was assessed in 111 patients. In the 35 patients with an urgent recommendation for KA by the IP (“as soon as possible” or “fixed date for KA”), the SP confirmed the current recommendation for immediate surgery in 13 patients (37%). In a further 16 patients (46%) he recommended a later KA if the condition worsened over time and in six patients (17%) he recommended no KA.

**Table 3:** Association between the recommendation of the initial physician and the second opinion physician (*n* = 111)

	Recommendation of the second opinion physician for knee arthroplasty			
	Yes	If the condition is worsening	No	Sum of the row n (% of all)
Urgency of recommendation for knee arthroplasty, initial physician, n (% from category)				
"fixed date for surgery”	5 (24%)	13 (62%)	3 (14%)	21 (19%)
"as soon as possible”	8 (57%)	3 (21%)	3 (21%)	14 (13%)
"can wait a few more months”	14 (56%)	9 (36%)	2 (8%)	25 (23%)
"if condition is worsening”	13 (42%)	16 (52%)	2 (7%)	31 (28%)
"at some point in time”	4 (20%)	7 (35%)	9 (45%)	20 (18%)
Sum of the column, n (% of all)	44 (40%)^a^	48 (43%)^a^	19 (17%)^a^	

^a^Frequencies of patients with available data for the urgency of the recommendation of the initial physician. The frequencies for all 141 patients for yes/if the condition is worsening/no are 40%/40%/20%

### Effects of the second opinion on the patient’s decision

**Fig. 2:**
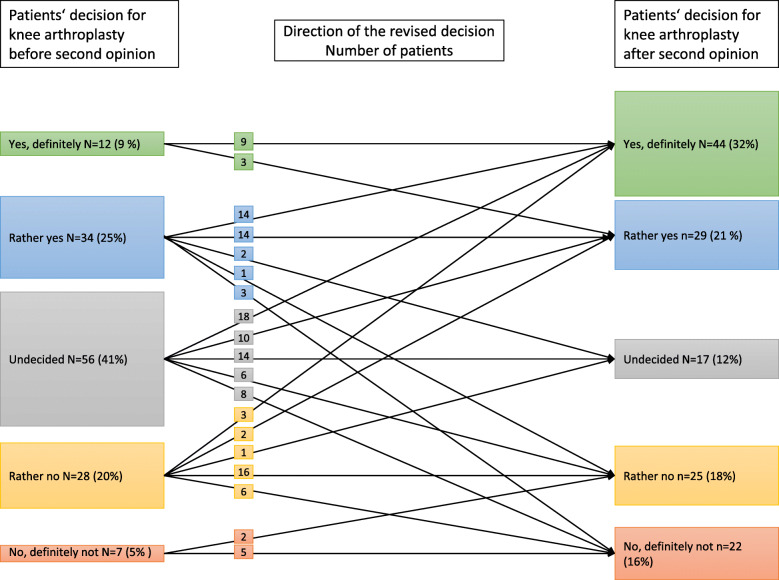
Change in the patients’ decision preference (*N* = 137)

Changes in the patients’ decisions between T0 and T1 are shown in Fig. 2 (total *N* = 137). The number of undecided patients decreased from 56 (41%) to 17 (12%) and the number of patients who were sure of their decision for or against KA increased from 19 (14%) to 66 (48%) (*p* < 0.001). The SO improved the decision confidence from 5.4 (± 3.0) to 7.8 (± 2.5) (p < 0.001).

The average influence of the SO on patients’ decisions was 1.79 (± 0.84) on a scale of 1–5. The influence was considered very strong by 56 (42%) and strong by 41 (43%) of 135 answering patients.

### Satisfaction with the second opinion concept

**Fig. 3:**
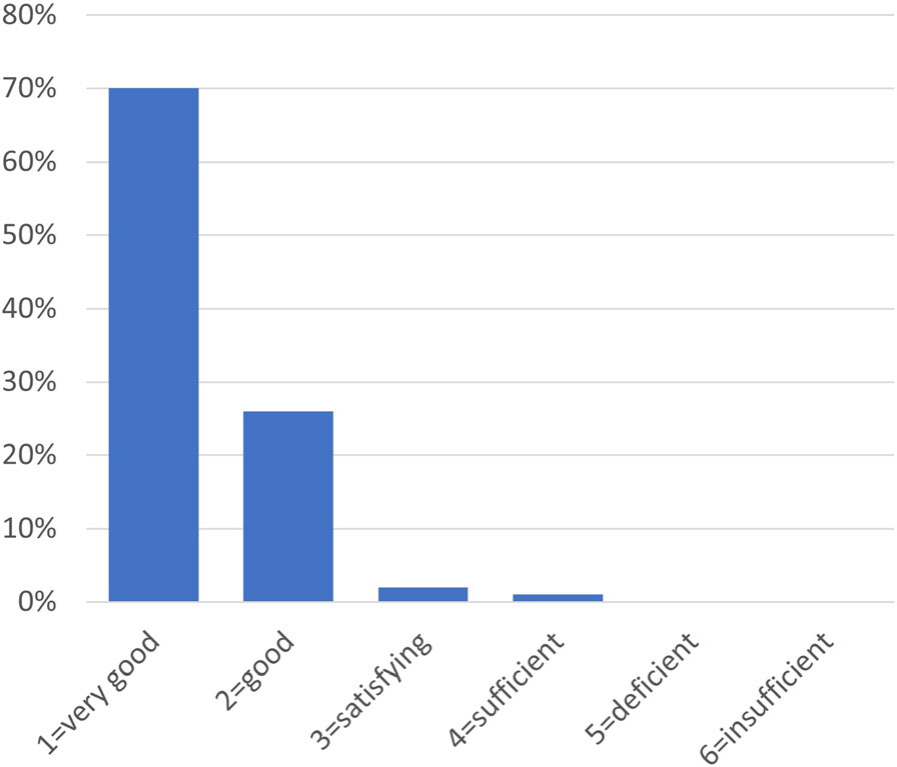
Patient satisfaction with the second opinion programme

The SO concept received an average school grade of 1.35 (± 0.60) on a scale from 1 to 6 (1 = best; 6 = worst). The distribution of the grades is presented in Fig. 3.

### Association between the recommendation of the SP and the indication criteria of the S2K guideline “Indication for KA”

Tables 1 and 2 compare the results of patients with and without a current recommendation for KA by the SP. Patients with a current recommendation for KA had higher radiological severity, higher pain intensity and lower knee-joint-specific quality of life. They more frequently reported previous exercise therapy and regular intake of pain medication.

In the logistic multivariable regression model (Table 4), a higher K-L score (*p* = 0.001) and lower knee-joint-specific quality of life (*p* = 0.041) predicted a recommendation for KA. The goodness-of-fit test of the logistic, multivariable regression model showed no indications for poor fit (χ^2^-Test = 3.908; *p* = 0.865).

**Table 4:** Multivariable logistic regression model, predictors for recommendation of knee arthroplasty by the second opinion physician

Criterion	Unadjusted odds ratio	Multivariable logistic regression			
		Adjusted odds ratio^d^	95% CI		*p*-value
Kellgren-Lawrence score^a^ (3 or 4 versus 1 or 2)	16.09	17.24	3.65	81.38	0.001
KOOS^b^ score – pain	1.01	0.99	0.95	1.02	0.486
KOOS^b^ score – quality of life	1.03	1.04	1.00	1.08	0.041
Exercise therapy^c^	1.89	1.88	0.63	5.57	0.258
Pain medication (reference: none)					0.089
On demand	0.73	0.38	0.12	1.21	0.101
Regular	1.50	1.10	0.31	3.91	0.873
Age – yr	1.02	1.03	0.98	1.08	0.234
Sex (reference: male)	1.09	1.02	0.41	2.53	0.975
Nagelkerkes *R*^2^: 0.346^e^					

^**a**^Kellgren-Lawrence score: range from 0 to 4, with higher scores indicating more severe disease. Scores of 1 and 2 as well as scores of 3 and 4 were combined

^**b**^KOOS: Knee Injury and Osteoarthritis Outcome Score, scores ranging from 0 (worst) to 100 (best)

^**c**^Exercise therapy: supervised strength or endurance training (individually or in group), home-based strength or endurance training or comprehensive rehabilitation intervention

^d^Adjusted odds ratio, meaning: Kellgren-Lawrence score^**a**^: 17.24 times higher probability for a recommendation of grade 3 or 4 than of grade 1 or 2; KOOS Quality of Life: Probability of a recommendation increases by 4% per unit increase;

^e^Nagelkerkes *R*^2^: The explained variance of the model was 34.6%

## Discussion

In this observational study, only 40% of cases initially recommended to have KA could be confirmed through the SO of an experienced knee surgeon in a certified knee arthroplasty centre after an in person consultation. In a further 40% of initial recommendations, a later KA was recommended if the condition worsens. In 20% of cases, the SP completely disagreed with the previous recommendation for KA. Predictors for the current recommendation for surgery by the SP physician were radiological severity of osteoarthritis and a lower knee-pain-specific quality of life. Both are main criteria for the indication of KA according to the German S2K guideline “Indication for KA”. These results suggest that the option of a SO with defined quality standards is important and may support treatment recommendations which adhere to guidelines. To obtain a more detailed picture of the association between the recommendation of the IP and the SP, we asked the patients for the urgency of the recommendation for KA by the IP. The SP was only able to confirm the current indication for KA in 24% of the patients to whom the IP had already recommended a fixed a date of surgery. An explanation for this low confirmation rate could be that after making the appointment for surgery, the patients may have read more information about KA and experienced doubts regarding the acute indication for KA based on this information. These doubts may have encouraged them to seek a SO. If the doubts of the patients were caused by guideline-conforming patient information, the SP may have not recommended KA.

The literature reports some reasons for discrepancies between the recommendations of an IP and SP. Financial incentives for KA implantation and surgery plannability as well as a limited budget for the prescription of exercise therapy are mentioned [9]. It has also been discussed that some patients and some physicians have negative attitudes towards conservative osteoarthritis treatment despite many guidelines with consistent evidence-based recommendations for exercise therapy, self-management programmes and weight loss in overweight patients [11, 31, 32]. They consider osteoarthritis as fateful and later surgery as inevitable [33].

To our knowledge, there is only one previous study that evaluated an SO programme with face-to-face contact that reports specific results for knee surgery [34]. That study was, however, conducted in 1978. Accordingly, the 52% of confirmed treatments reported in the study are not comparable to present SO programmes as the surgical techniques and guidelines have changed significantly since then.

The 40% agreement rate between the IP and SP for a current indication for KA identified in our study was higher than that from another evaluation of an SO study in Germany. That study found only 26% agreement between the IP and SP for the recommendation of knee surgery [35, 36]. However, and in contrast to the present study, the SP did not see patients in person, and specific results for KA were not presented. In the national SO programme “Best Doctors, Inc” in the United States, 34.6% of orthopaedic surgery patients changed their treatment after the SP recommendation [37]. However, again, patients were not seen personally by the SP, and no specific data were reported for patients with KA.

The frequency of patients (85%) who reported a strong or very strong influence of the SO on the treatment decision in this study was even higher than the 60 and 61% of patients reported in the literature [36, 37]. The face-to-face contact with the SP may have contributed to the high influence of the SO on the treatment preference. The strong influence of the SO on treatment decisions emphasises the need to define high-quality standards for the SO in patients before elective surgery.

The SO substantially reduced the percentage of undecided patients. In contrast, few patients who had preferences for or against KA before the SO changed their preferences, and only very few patients with preferences before the SO were undecided after the SO. These results suggest that in particular patients with decisional conflicts benefit from the SO. Furthermore, it contradicts concerns that different opinions between IP and SP cause decisional conflicts in patients.

In accordance with the guidelines, radiological criteria and knee-joint-specific quality of life were significant predictors for the recommendation of KA by the SP. These positive effects of the SO on adherence to guidelines have not yet been proven for elective surgery, while the avoidance of errors through a SO in pathology and radiology is already well documented [38, 39].

New evidence-based guidelines strongly recommend weight reduction in overweight people in addition to exercise therapy and self-management programmes for patients with osteoarthritis of the knee [31, 32]. This study showed that the pre-operative treatment of patients with recommendations for KA did not consistently comply with these guidelines. Almost a quarter of the patients did not receive active exercise therapy, and only 7% received recommendations or therapies for weight reduction, although 36% of patients were obese.

The quality of a decision is considered high if the patient is well informed, the recommended treatment is clinically appropriate, and the treatment meets the goal of the patient [40]. The influence of the SO on the decision and the high confidence in the decision indicate that the SO concept improves the first criterion, and the concordance of the recommendation of the SP with criteria from the guideline supports the improvement of the second criterion.

The high satisfaction of the patients with 97% of patients rating the SO concept “very good” or “good” is in line with the results from SO portals who report 89 to 95% of satisfied patients [35–37].

The main strength of the present study is that, to the best of our knowledge, it is the first study that systematically analyses the effects of an SO prior to KA. It is also the first study that evaluated an SO that is in concordance with the quality criteria for SOs that are required by the German Code of Social Law V, § 27b [13].

### Limitations

This study has some limitations. Firstly, the lack of data from non-participants could result in a self-selection of patients with more optimistic results. However, we are confident that this may introduce little or no bias because the non-participants were comparable in age and gender and the willingness to participate was higher than in comparable studies [34, 37]. Secondly, the patients were not followed further. Thus, in this study, we could not confirm that patients finally followed their intended decision. However, other studies suggest that the majority of patients adhere to their decision after the SO [36]. Thirdly, the SP did know the recommendations for KA made by the IP. This could increase the frequency of recommendations for KA because some studies on SOs showed that an SP who knows about a recommendation for interventional therapy by an IP tends to recommend interventional therapy more often than those who do not know the initial therapy. In contrast, the frequency of recommendations for KA in this study could be reduced by the fact that the SP was reimbursed by the health insurance AOK that may be interested in saving costs by reducing the frequency of KA recommendations. However, AOK did not provide any incentives to reduce the frequency of KA recommendations.

As in previous studies on SO, a different time between the initial opinion and the SO is a possible confounder that was not measured. A delay of the SP may reduce disagreement between the IP and the SP because the criteria knee pain, quality of life and previous conservative treatment have a tendency for getting worse over time in patients with OA. However, a major effect of time on disagreement rates seems unlikely because the SP considered the status in pain and quality of live in the last 3 to 6 months as recommended in guidelines. In addition, the delay could not introduce bias to the criterion structural damage because the SP assessed the same x-rays as the IP. However, future studies should address this potential source of bias.

Lastly, patients who seek a SO are likely different in their characteristics from patients who do not seek a SO. Higher disagreement in SO programmes with patients who voluntarily sought a SO compared to SO programmes were all patients have to present to a SP strongly support this hypothesis [34]. One obvious reason that may explain higher disagreement rates in voluntarily SO programmes are reasonable doubts of the patients that motivate them to seek a SO. Accordingly, the results of our study should not be generalized to all patients with a recommendation for KA.

## Conclusions

SO concepts can have a great influence on a patient’s therapy decision. A SO with personal presentation to an experienced knee surgeon in a certified knee arthroplasty centre may improve adherence to the guidelines for KA indications. From the patient’s perspective, a SO can reduce uncertainty and improve confidence in the treatment decision. Future studies, preferably with control group designs, should evaluate reasons for seeking a SO and predictive factors for differences between the first opinion and the SO.

## Data Availability

The analysis plan is available at https://osf.io/. Project name: “Evaluation Zweitmeinung Knieprothese”. The data set is not available because patients did not consent to the use of their data in a public repository.

## Abbreviations

KA: Knee arthroplasty
AWMF: Association of the Scientific Medical Societies in Germany
SO: Second medical opinion
SP: Second opinion physician
G-BA: Federal Joint Committee (German: Gemeinsamer Bundesausschuss)
IP: Initial physician
OPMR: Department of Orthopaedics, Physical Medicine and Rehabilitation
K-L: Kellgren and Lawrence
KOOS: Knee Injury and Osteoarthritis Outcome Score
EQ-5D-5L: 5-level version of the EuroQOL Group 5-Dimension Self-Report Questionnaire
PHQ-4: Patient Health Questionnaire 4
